# Influence of combined CYP2C19 and CYP2D6 phenotypes on adverse drug reactions in patients with major depressive disorder: a clinical cohort study

**DOI:** 10.1038/s41397-026-00407-3

**Published:** 2026-04-09

**Authors:** Carolin Görnert, Maike Scherf-Clavel, Heike Weber, Sibylle C. Roll, Andreas Eckert, Andreas Reif, Martina Hahn

**Affiliations:** 1 Department of Psychiatry, Psychosomatics and Psychotherapy, University Hospital Frankfurt, Goethe University, Heinrich-Hoffmann-Straße 10, 60528 Frankfurt am Main, Germany; 2 Department of Mental Health, varisano Hospital Frankfurt Höchst, Gotenstraße 6-8, 65929 Frankfurt am Main, Germany; 3 Department of Psychiatry, Psychosomatics and Psychotherapy, University Hospital Würzburg, Margarete-Höppel-Platz 1, 97080 Würzburg, Germany; 4 Fraunhofer Institute for Translational Medicine and Pharmacology ITMP, Theodor-Stern-Kai 7, 60596 Frankfurt am Main, Germany

**Keywords:** Risk factors, Genotype, Drug safety, Pharmacogenetics, Depression

## Abstract

Variants in cytochrome P450 enzymes are known risk factors for developing adverse drug reactions (ADR). Most antidepressants (AD) are simultaneously metabolized by major or minor pathway of CYP2C19 and CYP2D6, resulting in a complex interplay of metabolites. This study is one of the first to investigate and demonstrate the combined CYP 2C19/2D6 functional metabolic status as a possible risk factor for ADR in AD treatment of major depressive disorder. Most prescribed AD venlafaxine underwent subgroup analysis. More ADR in non-normal metabolizers (nNM) for one or both CYP enzymes compared with normal metabolizers (*p* = 0.039) were observed. Both slow (PM and IM) and rapid metabolizers (RM and UM) were affected. There were non-significant trends for CYP2C19 RM and UM with ADR in venlafaxine, which may be avoided in CYP2C19 nNM. More research is required to identify risk variants for personalized and safe AD treatment.

## Introduction

Treatment with antidepressants (AD) may go along with poor efficacy and tolerability [1]. Almost half of adverse drug reactions (ADR) can be attributed to interindividual variations in hepatic drug metabolism [2]. At least 20% of these ADR are preventable [3]. To enhance safety of drug therapy, the Clinical Pharmacogenomics Implementation Consortium (CPIC) and the Dutch Pharmacogenetics Working Group (DPWG) have published pharmacogenetic-guided decision support tools. These guidelines provide dosage and action recommendations for prescribing certain AD based on CYP genotypes [4–7]. The two highly polymorphic cytochrome P450 isoenzymes CYP2C19 and CYP2D6 are particularly important in the metabolism of AD [8–10]. Divergent (= non-normal-metabolizer (nNM)) genotypes are common in patients suffering from severe mental disorders [11, 12].

Isolated studies indicate that poor metabolizers of CYP2C19 or CYP2D6 experience more ADR on AD therapy than normal metabolizers [13–18]. This is attributed to increased serum concentrations of the active drug moieties [19–21]. Rapid CYP2C19 or CYP2D6 metabolizers have been associated with insufficient clinical response and drug discontinuation due to sub-therapeutic serum concentrations [22, 23]. An increased risk of ADR has not been demonstrated for UM [13, 24].

Although phenoconversion (PC) occurs in up to 44.9% of patients undergoing psychopharmacotherapy, studies have often utilized CYP genotype instead of phenotype [13, 18, 25, 26]. Inhibitors decrease the CYP activity, leading to higher drug concentrations of the substrate [27–30]. In addition to these drug-gene interactions, drug-gene-gene interactions (DDGI) may also occur because the metabolism of certain AD, such as venlafaxine (VEN), is catalyzed by several CYP isoenzymes [31]. The development of multiplex genotyping devices allowed examination of combined CYP450 genotypes [32]. The first multigene genotype score for CYP2C9, CYP2C19, and CYP2D6 was published in 2011, allowing drug metabolic indices to be derived [33]. Impaired CYP enzyme activity can lead to ADR and activation of an alternative pathway, altering serum levels of the parent drug, metabolites, and parent-metabolite ratios [34]. Studies have investigated serum concentrations in relation to two CYP isoenzyme phenotypes, but the impact on clinical tolerability is limited [35–37]. The influence of combined functional CYP 2C19/2D6 enzyme status on VEN tolerability has only been investigated in case studies [38–40]. There are also no controlled studies on tolerability based on combined CYP isoenzymes for most other AD, except for amitriptyline, where combined pharmacogenomic (PGx) testing for CYP 2C19/2D6 revealed risk constellations for ADR [14].

PGx-guided therapies can lead to better response, faster remission rates, lower incidence of ADR, and thus significant long-term cost savings for healthcare system [41–46]. A better understanding of gene-gene interaction of functional CYP 2C19/2D6 enzyme status on AD tolerability may contribute to an improved therapy of major depressive disorder (MDD). Cross-tabulations based on combined phenotypes of CYP 2C19/2D6 for amitriptyline and CYP 2B6/2C19 for sertraline have already been published by CPIC [4, 5].

This study investigates the frequency and intensity of ADR during AD therapy in a cohort of hospitalized psychiatric patients regarding their CYP2C19 and CYP2D6 functional status. Pharmacodynamic and pharmacokinetic factors were considered. Furthermore, a drug-specific subgroup analysis was performed for VEN.

## Method

### Study design

104 adult patients voluntarily admitted to the Department of Psychiatry at the University Hospital Frankfurt were recruited for the FACT-PGx clinical cohort study between July 2021 and July 2022. The sample size was chosen because rare genotypes CYP2D6 UM and CYP2C19 PM statistically occur at least once in 100 European Caucasian individuals, allowing all different genotypes to be included in the study.

Patients were treated for MDD according to ICD-10 F32.x and F33.x criteria, regardless of whether AD or other psychotropics had been taken at the time of admission. There were no restrictions on ethnicity and choice, number, or dosage of AD prescribed.

Patients were asked to complete the subjective questionnaire “*SeIf-Rating Scale for Adverse Drug Effects*“ developed by Dreher et al. for AD treatment [47]. It examines 16 defined and up to two individual ADR based on their incidence (yes/no), intensity (not disturbing/unpleasant/very unpleasant/unbearable), and likelihood of a causal drug effect (probable/possible/improbable). ADR reported as unlikely to be medication-related were corrected for further analysis. Patients who did not complete the questionnaire or submitted it incompletely were excluded from the analysis (*n* = 49). Data collection was one-pointed. The clinical response to AD typically occurs within 14 days, and initial ADR usually subside after this period [48]. Therefore, only patients who had not taken a new AD in the previous 14 days were considered for statistical analysis of stable ADR (*n* = 41). We evaluated ADR depending on functional CYP enzyme status, considering only patients from this group whose AD are mainly metabolized by CYP2C19 and/or CYP2D6 (*n* = 35).

On the same day, blood was drawn for genotyping. Genotyping was performed in the laboratory of the Department of Psychiatry at University Hospital Würzburg, which was certified by a quality control program [49]. DNA was isolated from EDTA blood samples, and relevant gene variants (single nucleotide polymorphisms) in CYP2C19 and CYP2D6 were genotyped using a MassArray Analyzer 4 system (Agena Bioscience GmbH, Hamburg, Germany). This process employed a self-designed panel utilizing SpectroCHIP®-96 Arrays and iPLEX® Pro chemistry, following the manufacturer’s instructions provided. Moreover, copy number variations (CNV) were determined using CYP2D6 RealFast™ CNV Assay [50]. Single nucleotide polymorphisms were translated into star alleles using the Pharmacogene Variation Consortium website (www.pharmvar.org). Haplotype tables were reported elsewhere [28, 30]. Each star allele was assigned an activity value based on CPIC definition table [5], and an activity score was calculated by summing all allelic activity values of the diplotype [51, 52]. Finally, the genetic phenotype was calculated by considering PC. The Flockhart table published by the Food and Drug Administration (FDA) was used to identify CYP enzyme-specific inducers and inhibitors in the current medication, and the genotype-predicted phenotype of CYP2C19 and CYP2D6 was calculated [31, 53, 54]. The inhibitor strength level of promethazine is currently under review. Therefore, it was not considered in PC calculation. All following analyses regarding CYP enzyme status were performed considering PC. Phenotypes were classified as PM (poor metabolizer), IM (intermediate metabolizer), NM (normal metabolizer), UM (ultrarapid metabolizer), and CYP2C19 additionally RM (rapid metabolizer) [55]. CYP2C19 UM and RM were grouped as rapid metabolizers (RM). Due to the small number of PM, these were grouped with IM as slow metabolizers (SM). The genotyping result was unknown to the participants when they completed the questionnaire.

All study participants gave informed consent. The study was approved by the Ethics Committee of the Goethe University Frankfurt (2021–138) and was conducted following the Declaration of Helsinki 2013.

### Statistical analyses

Data were collected, processed, and analyzed descriptively and statistically using Microsoft Excel version 16.66.1. Independent t-tests were performed to analyze continuous parametric discrete variables, while chi-square tests were used to analyze categorical dichotomous variables. A P-value of < 0.05 (*) was considered significant. P-values were adjusted for multiple testing using the Benjamini-Hochberg False Discovery Rate (FDR) correction.

An Odds Ratio (OR [95% confidence interval, CI]) greater than 1 indicated that the ADR was more likely to occur in the exposure group. Empty cells for specific ADR were replaced by adding 0.5 (Haldane-Anscombe correction) to calculate OR [56]. To avoid distortion of statistical evidence, significance tests were not performed in this case. Data are presented as mean ± standard deviation unless specified otherwise.

Linear regression analyses were performed to explore associations between ADR and pharmacogenetic as well as demographic variables (Supplementary Tables S1–S2). Owing to the limited sample size (*n* = 35), these analyses were conducted on an exploratory basis. Separate linear regression analyses for venlafaxine were not performed due to insufficient sample size (*n* = 12), precluding reliable parameter estimation.

## Results

### Patient characteristics

55 of 104 genotyped patients (52.9%) completed the questionnaire. Of these patients, 35 had been taking an AD metabolized by either CYP2C19 and/or CYP2D6 for at least two weeks. Their mean age was 41.5 years (±15, range 19–77), with 62.9% identifying as female and 37.1% as current smokers. In the context of a first depressive episode, 14.3% were first hospitalized and treatment naïve. AD were primarily used as monotherapies (82.9%), with VEN (*n* = 12), sertraline (*n* = 8), and escitalopram (*n* = 7) being administered most frequently. Augmentation with second or third-generation antipsychotics (*n* = 11) or lithium (*n* = 3) occurred in 40.0%. Demographic and drug use characteristics of responding versus not submitting patients are shown in Table 1. The drop-out group included a higher number of RM for both CYP isoenzymes and more males, while treatment with selective serotonin reuptake inhibitors (SSRI) was less common.

**Table 1:** Characteristics of patients whose antidepressants are mainly metabolized by CYP2C19 and/or CYP2D6 (*n* = 35, “responders”) in contrast to patients who did not submit the questionnaire (*n* = 49, “drop-outs”).

Characteristics	Responses	Total (%) of responders *n* = 35	Total (%) of drop-outs *n* = 49
Age		41.5 ± 15.0 years, range 19-77	45.4 ± 15.7 years, range 21-73
Sex	Male	13 (37.1)	29 (59.2)
	Female	22 (62.9)	21 (42.8)
Smoking status	Smoker	13 (37.1)	23 (46.9)
	Non-smoker	22 (62.9)	26 (53.1)
Clinical parameters	Renal impairment (GFR < 60 ml/min)	0 (0.0)	1 (2.0)
	Hepatic impairment (liver enzymes 3-fold upper normal limits)	0 (0.0)	0 (0.0)
Diagnosis according to ICD-10	Depressive episode (ICD-10 F32.x)	5 (14.3)	8 (16.3)
	Recurrent depressive disorder (ICD-10 F33.x)	30 (85.7)	41 (83.7)
Antidepressant medication	Selective Serotonin Reuptake Inhibitors (SSRI)	18 (51.4)	13 (26.5)
	Sertraline (*n* = 8 / 10)		
	Escitalopram (*n* = 7 / 3)		
	Fluoxetine (*n* = 3 / 0)		
	Serotonin Noradrenalin Reuptake Inhibitors (SNRI)	14 (40.0)	17 (34.7)
	Venlafaxine (*n* = 12 / 16)		
	Duloxetine (*n* = 2 / 1)		
	Alpha-2-Antagonist (A2A)	7 (20.0)	10 (20.4)
	Mirtazapine (*n* = 7 / 10)		
	Tricyclic Antidepressants (TCA)	2 (5.7)	6 (12.2)
	Amitriptyline (*n* = 1 / 5)		
	Clomipramine (*n* = 1 / 0)		
	Trimipramine (*n* = 0 / 1)		
	Not mainly metabolized by CYP2C19/CYP2D6	1 (2.9)	0 (0.0)
	Bupropion (*n* = 1 / 0)		
Form of antidepressant treatment	Monotherapy	29 (82.9)	44 (89.8)
	Combination with other antidepressants	6 (17.1)	5 (1.0)
	Augmentation with second or third-generation antipsychotics or lithium	14 (40.0)	24 (49.0)
CYP enzyme status			
Genotype-predicted CYP2C19 phenotype	NM	13 (37.1)	21 (42.9)
	IM	12 (34.3)	8 (16.3)
	PM	0 (0.0)	1 (2.0)
	RM	7 (20.0)	17 (34.7)
	UM	3 (8.6)	2 (4.1)
Genotype-predicted CYP2D6 phenotype	NM	23 (65.7)	23 (46.9)
	IM	10 (28.7)	18 (36.7)
	PM	1 (2.9)	5 (10.2)
	UM	1 (2.9)	3 (6.1)
Phenoconversion-predicted CYP2C19 phenotype	NM	12 (34.3)	21 (42.9)
	IM	11 (31.4)	8 (16.3)
	PM	2 (5.7)	1 (2.0)
	RM	7 (20.0)	17 (34.7)
	UM	3 (8.6)	2 (4.1)
Phenoconversion-predicted CYP2D6 phenotype	NM	11 (31.4)	22 (44.9)
	IM	19 (54.3)	19 (38.8)
	PM	5 (14.3)	5 (10.2)
	UM	0 (0.0)	3 (6.1)

**Fig. 1:**
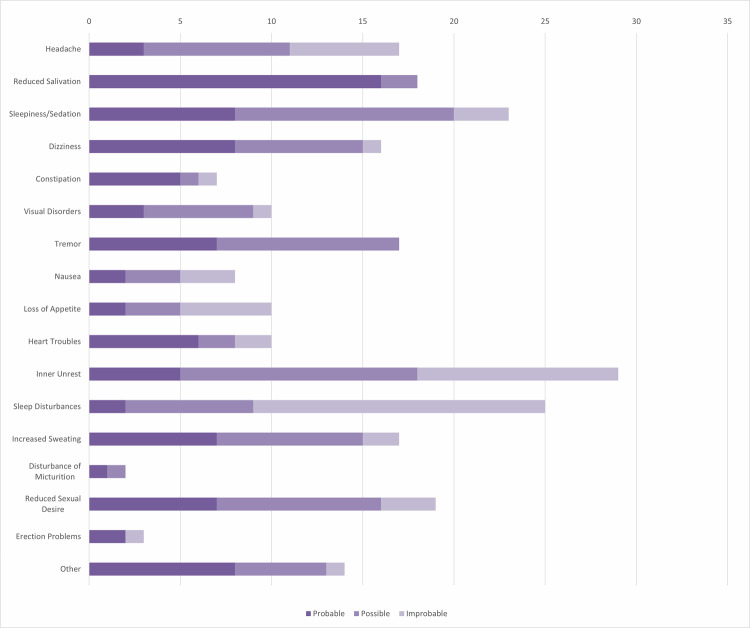
Overview of ADR incidence as reported in the *“SeIf-Rating Scale for Adverse Drug Effects”* by Dreher et al. Patients rated the causality of ADR with their prescribed antidepressants (*n* = 35).

As illustrated in Fig. 1, patients were asked to rate the causality of AD for each symptom. The three most prevalent ADR were inner unrest (*n* = 30, 85.7%), sleep disturbances (*n* = 27, 77.1%), and sleepiness (*n* = 26, 74.4%), which, according to DSM-5, are similar to characteristic symptoms of depression [57]. Following the exclusion of ADR that were considered unlikely to be medication-related by the patients, most frequently reported symptoms were still sleepiness (*n* = 23, 65.7%), reduced salivation (*n* = 20, 57.1%), inner unrest and tremor (both *n* = 19, 54.3%). The group reported suffering from an average of 5.9 (±3.2) ADR. Of these, 11.7% were classified as not disturbing, 47.3% as unpleasant, 33.2% as very unpleasant, and 6.8% as unbearable.

### CYP2C19 and CYP2D6 phenotypes

For CYP2D6 and CYP2C19, we observed a high PC rate (*n* = 17, 48.57%) in the context of concomitant use of perpetrator drugs (Table 2).

**Table 2:** Perpetrator drugs on the CYP2D6 and CYP2C19 genotype-predicted phenotype (*n* = 35).

Perpetrator drugs on the CYP2D6 genotype	Total (%)
Inhibitors	33 (73.3)
Sertraline^3^	9
Escitalopram^3^	7
Bupropion^1^	4
Fluoxetine^1^	3
Duloxetine^1^	2
Promethazine^4^	8
**Perpetrator drugs on the CYP2C19 genotype**	
Inhibitors	11 (24.4)
Pantoprazole^3^	6
Fluoxetine^2^	4
Oral contraceptive^3^	1
Inducer	1 (2.2)
Prednisolone	1

^1^Strong inhibitor: causes a > 5-fold increase in the plasma area under the curve (AUC) values or more than 80% decrease in clearance; ^2^Moderate inhibitor: causes a > 2-fold increase in the plasma AUC values or 50-80% decrease in clearance; ^3^Weak inhibitor: causes a > 1.25-fold but < 2-fold increase in the plasma AUC values or 20-50% decrease in clearance; ^4^Inhibitor strength level is under review.

Source: The Flockhart Cytochrome P450 Drug-Drug Interaction Table.

CYP2D6 was most frequently affected (*n* = 14). Inhibition led to a twofold rise in the proportion of CYP2D6 SM, from 31.9–68.57%. CYP2D6 UM could no longer be observed after PC. CYP2C19 genotype exhibited minimal susceptibility to DDGI, resulting in no alteration in the number of SM and RM. All following analyses and figures regarding CYP enzyme status were performed considering PC. Phenotype combinations occurring in the study population were cross-tabulated in a combinational table similar to those used in CPIC guidelines (Table 3). The combination CYP 2C19/2D6 NM/NM occurred three times and served as the reference group for calculating the OR. OR for specific ADR in nNM compared to NM are shown in Table 4.

**Table 3:** Observed CYP 2C19/2D6 phenotypes predicted following phenoconversion (*n* = 35).

Phenotype		CYP2D6			
		PM *n* (%)	IM *n* (%)	NM *n* (%)	UM *n* (%)
**CYP2C19**	**PM** *n* (%)	2 (5.7)	-	-	-
	**IM** *n* (%)	1 (2.9)	6 (17.1)	4 (11.4)	-
	**NM** *n* (%)	2 (5.7)	7 (20.0)	3 (8.6)	-
	**RM** *n* (%)	-	4 (11.4)	3 (8.6)	-
	**UM** *n* (%)	-	2 (5.7)	1 (2.9)	-

**Table 4:** Odds Ratio (95% CI) for ADR of phenoconversion predicted NM/NM phenotype compared to non-NM (*n* = 35).

Adverse side effect	Phenotype		
	CYP2C19/CYP2D6	CYP2C19	CYP2D6
Headache	0.91 (0.07–11.23)	0.88 (0.20–3.89)	**1.33** (0.28–6.44)
Reduced Salivation	**2.92** (0.24–35.68)	0.93 (0.23–3.82)	0.68 (0.16–2.93)
Sleepiness/Sedation	**4.40** (0.36–54.37)	0.94 (0.21–4.10)	**1.14** (0.26–5.09)
Dizziness	**7.00** (0.33–146.45)	0.46 (0.11–1.90)	**1.75** (0.40–7.58)
Constipation	0.46 (0.04–5.97)	**3.88** (0.41–36.79)	0.25 (0.04–1.40)
Visual Disorders	**2.83** (0.13–60.2)	**5.87** (0.64–54.00)	0.46 (0.10–2.22)
Tremor	**2.57** (0.21–31.33)	**2.18** (0.53–9.02)	0.57 (0.13–2.48)
Nausea	**1.72** (0.08–37.54)	**1.05** (0.16–6.78)	0.90 (0.14–5.84)
Loss of Appetite	**1.40** (0.06–31.12)	0.75 (0.11–5.24)	0.64 (0.09–4.53)
Heart Troubles	**2.43** (0.11–52.01)	0.83 (0.16–4.30)	**1.50** (0.25–8.98)
Inner Unrest	**10.11** (0.48–212.09)	0.78 (0.19–3.19)	**1.68** (0.40–7.07)
Sleep Disturbances	**3.74** (0.18–78.94)	**1.60** (0.34–7.64)	**2.70** (0.47–15.40)
Increased Sweating	**7.00** (0.33–146.45)	**2.18** 0.51–9.33)	**1.02** (0.24–4.26)
Disturbance of Micturition	0.57 (0.02–14.53)	**2.91** (0.13–65.53)	0.43 (0.02–7.66)
Reduced Sexual Desire	0.50 (0.04–6.08)	0.21 (0.05–1.01)	**2.45** (0.56–10.68)
Erection Problems	0.57 (0.02–14.53)	**2.91** (0.13–65.53)	**2.56** (0.11–57.78)
Other	**1.15** (0.12–10.8)	**1.22** (0.33–4.45)	0.46 (0.13–1.56)
**Total**	2.99* (**1.38–6.46)**	**1.11** (**0.78–1.58)**	0.98 (0.69–1.40)

The bolded values indicate that the odds ratio is higher in the non-NM phenotype risk group than in the NM/NM wild-type variant group

**Fig. 2:**
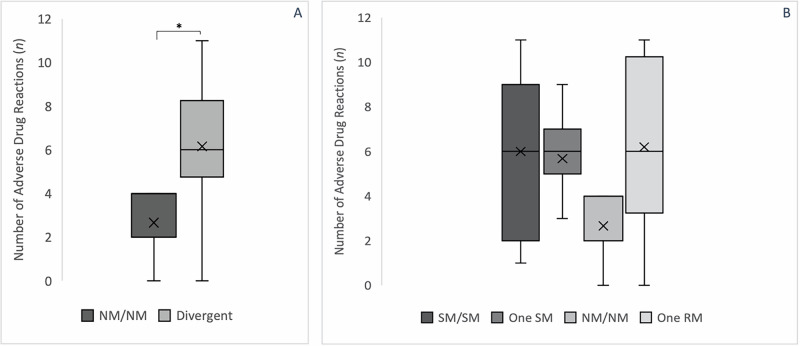
Number of ADR depending on the combined phenoconversion predicted CYP 2C19/2D6 phenotype (*n* = 35). Combined CYP 2C19/2D6 phenotypes predicted following phenoconversion are shown. Compared to combined NM/NM, divergent phenotypes had a significantly higher number of ADR. **A** NM/NM (median: 4, IQR: 2) vs divergent phenotypes (median: 6, IQR: 3.5), p = 0.039*; (**B**) SM/SM (median: 6, IQR: 7) vs one SM (median: 6, IQR: 2) vs NM/NM (median: 4, IQR: 2) vs one RM (median: 6, IQR: 7). Mean (x); median (−); *p < 0.05; SM Slow Metabolizers (PM, IM); NM Normal Metabolizers; RM Rapid Metabolizers (RM, UM).

Functional nNM for CYP2C19 and CYP2D6 had an average of 6.2 (±3.2) ADR. This was 2.3 times more common than in NM/NM (2.7 ± 1.9) with an OR of 2.99 (CI 1.38-6.46, *p* = 0.039*) (Fig. 2**)**. ADR were observed more frequently with both slow (6.1 ± 2.8) and fast (6.2 ± 3.8) combined partners. The combination CYP 2C19/2D6 NM/IM or IM/NM already increased the OR for ADR (OR = 3.19, CI 1.39–7.36, *p* = 0.006*; OR = 2.53, CI 1.03–6.24, *p* = 0.166). ADR risk was highest for CYP 2C19/2D6 IM/PM and UM/NM (OR = 9.04, CI 2.7–30.27, *p* = 0.044*). Due to autoinhibition, PM/PM was observed twice when fluoxetine was taken, but it was not associated with a higher number of ADR (OR = 0.52, CI 0.13–2.12). After FDR correction, only the elevated ADR risk in CYP 2C19/2D6 NM/IM remained statistically significant.

Exploratory linear regression analyses, including CYP2C19 and CYP2D6, did not identify statistically significant predictors of ADR. Trends suggested a positive association between CYP2C19 metabolic activity and ADR frequency and an inverse association for CYP2D6. ADR increase was also observed with combined antidepressant therapy, higher age, and male sex (Supplementary Tables S1-S2).

For individual ADR, such as inner unrest (OR = 10.11, CI 0.48–212.09), increased sweating (OR = 7.00, CI 0.33–146.45), and sleepiness (OR = 4.40, CI 0.36–54.37), OR was notably increased in combination with a nNM for CYP2C19 or CYP2D6. CYP2C19 SM were associated with visual disturbances (OR = 5.87, CI 0.64–54.00).

### Venlafaxine subgroup

VEN was the most prescribed AD in the cohort. In the drug-specific analysis for VEN (*n* = 12), the appearance of a nNM, whether for CYP2C19 or CYP2D6, reported on average 4.4 more ADR (OR = 4.41, CI 1.49–13.04, *p* = 0.108) (Table 5). In particular, reduced salivation (OR = 10.71, CI 0.4–287.83), increased sweating, and inner unrest (both OR = 7.22, CI 0.28–189.19) occurred more frequently in this constellation compared to NM. Descriptively, only CYP2C19 nNM status influenced increased ADR with VEN, especially RM (OR = 1.30, CI 0.70–2.42, *p* = 0.356). CYP2D6 nNM occurred only as IM, an actionable genotype (AG) according to DPWG (Table 6), which had no effect on ADR (OR = 0.87, CI 0.44–1.69, *p* = 0.448).

**Table 5:** Odds Ratio (95% CI) for ADR of phenoconversion predicted NM/NM phenotype compared to non-NM for the venlafaxine subgroup (*n* = 12).

Adverse side effect	Phenotype		
	CYP2C19/CYP2D6	CYP2C19	CYP2D6
Headache	0.25 (0.01–5.98)	1.00 (0.06–15.99)	0.27 (0.01–6.74)
Reduced Salivation	**10.71** (0.4–287.83)	**1.67** (0.15–18.87)	**1.60** (0.10–24.70)
Sleepiness/Sedation	**1.50** (0.07–31.57)	**1.67** (0.15–18.87)	0.25 (0.02–4.00)
Dizziness	**3.46** (0.13–90.68)	**1.80** (0.12–26.20)	1.00 (0.06–15.99)
Constipation	**1.47** (0.05–41.83)	**3.46** (0.13–90.68)	0.40 (0.01–10.81)
Visual Disorders	**3.46** (0.13–90.68)	**1.80** (0.12–26.20)	1.00 (0.06–15.99)
Tremor	**1.50** (0.07–31.57)	**1.67** (0.15–18.87)	0.25 (0.02–4.00)
Nausea	0.79 (0.02–25.90)	**1.80** (0.06–54,33)	0.81 (0.03–25.10)
Loss of Appetite	**2.33** (0.09–62.68)	1.00 (0.06–15.99)	**1.75** (0.10–30.84)
Heart Troubles	**2.33** (0.09–62.68)	1.00 (0.06–15.99)	**1.75** (0.10–30.84)
Inner Unrest	**7.22** (0.28–189.19)	1.00 (0.09–11.03)	**2.50** (0.16–38.60)
Sleep Disturbances	**2.33** (0.09–62.68)	1.00 (0.06–15.99)	**1.75** (0.10–30.84)
Increased Sweating	**7.22** (0.28–189.19)	1.00 (0.09–11.03)	**2.50** (0.16–38.60)
Disturbance of Micturition	0.79 (0.02–25.9)	**1.80** (0.06–54.33)	0.81 (0.03–25.10)
Reduced Sexual Desire	0.43 (0.02–9.36)	0.33 (0.03–4.19)	1.00 (0.06–15.99)
Erection Problems	0.24 (0–15.19)	0.53 (0.01–31.41)	**2.71** (0.04–164.94)
Other	**5.00** (0.24–106.11)	**1.36** (0.20–9.28)	1.00 (0.14–7.10)
**Total**	**4.41** (**1.49–13.04)**	**1.30** (**0.70–2.42)**	0.87 (0.44–1.69)

**Table 6:** Actionable CYP-enzyme phenotypes and dose recommendations based on CPIC and DPWG guidelines.

Antidepressant	Phenotype	Total of participants (OR, 95% CI)	Guideline recommendation	References
Venlafaxine	CYP2D6 PM	-	Avoid or reduce dose	Bousman et al. [5] Beunk et al. [7]
	CYP2D6 IM	3 (0.87, CI 0.44–1.69)	Avoid or reduce dose	Beunk et al. [7]
Tricyclic antidepressants	CYP2D6 PM	-	Avoid or reduce dose by 50% and use therapeutic drug monitoring (TDM) to adjust dosing	Hicks et al. [4]
	CYP2D6 IM	1 (1.3, CI 0.31–5.39)	Reduce dose by 25% and use TDM to adjust dosing	
	CYP2D6 UM	-	Avoid or use TDM to adjust dosing	
	CYP2C19 PM	-	Avoid or reduce dose by 50% and use TDM to adjust dosing	
	CYP2C19 RM and UM	-	Avoid or use TDM to adjust dosing	
Citalopram Escitalopram	CYP2C19 PM	-	Select another antidepressant not predominantly metabolized by CYP2C19 or reduce dose by 50%	Bousman et al. [5]
	CYP2C19 UM	-	Select another antidepressant not predominantly metabolized by CYP2C19	
Sertraline	CYP2C19 PM	-	Reduce dose by 50%	Bousman et al. [5]
	CYP2B6 PM	-	Select another antidepressant not predominantly metabolized by CYP2B6 or reduce dose by 25%	

Besides CYP2D6 IM in VEN, our cohort included with CYP2D6 IM one AG for clomipramine, showing more ADR (OR = 1.3, CI 0.31–5.39). There were no AG for SSRI.

## Discussion

This study shows that AD treatment is highly associated with ADR, which are perceived as severe in over 40% of cases. Identifying individual risk factors is essential for preventing ADR and enhancing depression treatment adherence.

The study suggests CYP 2C19 and 2D6 nNM phenotype variants may be associated with an increased risk of ADR to psychotropic drugs. Particularly for CYP2C19, an elevated ADR risk was observed for both SM and RM. Consistent with previous studies, CYP2C19 SM are associated with intolerances due to increased serum concentrations [13].

To our knowledge, this study is the first to demonstrate that individuals who are RM may also exhibit an elevated number of ADR. This divergent observation may be attributable to DDGI and PC, which have not been adequately considered in numerous studies [13]. Given the accelerated degradation of AD and the resulting lower serum concentration, it appears counterintuitive that RM are more susceptible to ADR. However, the CYP system is complex, with many CYP isoenzymes involved in the degradation of an active substance. For instance, VEN is degraded to 90% via CYP2D6 and 10% via CYP2C19 in O-desmethyl-venlafaxine (ODV). Surprisingly, CYP2C19 RM had a greater effect on ADR incidence than CYP2D6. An alternative metabolic pathway for VEN involves N-demethylation by CYP2C19 and CYP3A4, resulting in N-desmethyl-venlafaxine (NDV), which has no antidepressant effect [58]. Accordingly, a rapid CYP2C19 phenotype could lead to a relative increase in NDV concentration. It is not sufficiently clear how the ratio of VEN to its metabolites and the stereoselectivity of the enantiomers contribute to ADR. Studies suggest that exposure to VEN, and possibly NDV, is more important for treatment tolerability than exposure to ODV [15, 59]. This may provide a rationale for increased ADR in CYP2C19 RM. Compared with ODV, VEN has a higher affinity for the noradrenergic system, which has been implicated in symptoms such as reduced salivation and increased sweating in CYP2D6 SM [60]. It is important to recognize that VEN serum concentration and effect cannot be explained by isolated CYP2C19 or CYP2D6 phenotype. A comprehensive consideration of both enzymes is essential [34]. Current guidelines for VEN dosing are limited to recommendations for CYP2D6 [5, 7]. CPIC and DPWG both advise against using VEN in PM, while DPWG also advises against its use in IM (Table 6). Both define IM as having a gene activity score of 0.25 through 1. The VEN subgroup contained no CYP2D6 PM, but IM. Our results did not show an increased ADR risk with IM compared to NM, which supports CPIC position using the standard dose. However, current guidelines do not provide any recommendations for VEN use in CYP2C19 nNM, which was identified as the primary cause of ADR in this study.

Furthermore, most AD lack therapy adjustment recommendations in RM or UM due to insufficient data evidence. An exception are tricyclic antidepressants (TCA), which should be avoided in UM for CYP2C19 or CYP2D6. In our findings, established AG alone do not fully explain ADR risk in AD treatment, underscoring the need for guidelines considering broader factors. Divergent combinations of CYP2C19 and CYP2D6 phenotypes appear to have additive adverse effects on AD pharmacokinetics, as shown in Tables 3 and 4 or in the previous example of VEN. This finding may also apply to other substance classes of AD.

PGx-guided dose recommendations are currently available from CPIC for most SSRI and TCA based on CYP2C19, CYP2D6, or CYP2B6 [4, 5]. While an isolated phenotype is usually considered, cross-tabulations already exist for amitriptyline (CYP 2C19/2D6) and sertraline (CYP 2B6/2C19) [4, 5]. Hicks et al. described CYP2C19 and CYP2D6 combined gene-based recommendations for amitriptyline with levels of recommendation of optional, moderate, and strong. TCA should be avoided in patients with CYP2D6 PM or UM and CYP2C19 PM. While amitriptyline therapy can be initiated without restriction when CYP2D6 in NM is considered alone (strong), it should be avoided in combination with CYP2C19 PM (moderate). Amitriptyline should also be avoided in CYP 2C19/2D6 NM/PM (severe) and NM/UM (severe). The authors note that clinical and pharmacokinetic data are currently lacking for stronger recommendations, mainly due to the rare occurrence of UM in previous studies. However, it can be deduced that extreme metabolizers such as PM and UM pose a risk for both CYP enzymes, as evidenced by pharmacokinetic observations of their metabolites. CYP2C19 metabolizes amitriptyline into active nortriptyline (NT), which is associated with a higher ADR prevalence than amitriptyline; the hydroxylated metabolites produced by CYP2D6 have a strong affinity for muscarinic receptors and have been associated with cardiotoxicity [61]. Steimer et al. hypothesized that CYP2C19 RM are linked with more ADR than CYP2C19 SM [14, 37]. Their study cohort had an absence of UM and a paucity of PM. They showed that CYP 2C19/2D6 IM/NM was the most beneficial combination for the tolerability of TCA.

Dosage recommendations are not available for all SSRI. There is no guideline for fluoxetine, as there is no evidence that CYP2C19 or CYP2D6 significantly impact clinical outcome [5]. The sum of active fluoxetine metabolites appears mainly independent of CYP2D6 metabolism status [62]. According to this, this study found no positive correlation with ADR in PM/PM. However, this group included only two patients genotyped for both enzymes as NM and only became PM/PM due to fluoxetine autoinhibition. Besides CYP2D6, CYP2C9 is significantly involved in fluoxetine metabolism without being autoinhibited by its substrate [63, 64]. CYP2C9 converts fluoxetine to R-norfluoxetine, which is less pharmacologically active than S-norfluoxetine produced by CYP2D6 [65]. Therefore, the CYP2C9 pathway could lead to a regular degradation of fluoxetine without an increased risk of ADR. This consideration is speculative, as our study did not include genotyping for CYP2C9. However, it highlights again that metabolism cannot be considered linear and that a gene panel is required to account for DGGI.

Besides amitriptyline and sertraline, combined gene-based recommendations have also been published by CPIC for other common drugs such as rosuvastatin (SLCO1B1/ABCG2) and fluvastatin (SLCO1B1/CYP2C9) to prevent statin-associated musculoskeletal symptoms and for thiopurines (TPMT/NUDT15) to prevent severe myelosuppression [66, 67]. A double-slow combination appears to be associated with additive negative pharmacokinetic effects. For example, fluvastatin is not recommended for individuals with CYP2C9 PM and reduced SLO1B1 function [66]. Combined genetic considerations also exist for warfarin, where CYP2C9 and VKORC1 genotypes have been associated with an increased risk of bleeding [68, 69].

Another risk factor for ADR is polypharmacy [70]. This can lead not only to drug-drug interactions but also to drug-gene interactions. Many psychotropic drugs are both substrates and inhibitors of CYP2D6 and CYP2C19. In our cohort, PC resulted in a deviation from the genetic phenotype in 48.57% of patients. This observation is representative of psychiatric inpatients [25, 26]. There is evidence that PGx-guided therapies reduce polypharmacy, making implementation even more important [44].

The study is limited by its small sample size due to excluded patients who did not complete the questionnaire. Explanations for this may include a disease-related decline in motivation or a possible linguistic barrier. A response rate of less than 50% is common in depression studies [71]. This may lead to non-response bias and misinterpretation of ADR prevalence, as patients experiencing more severe or milder ADR are less likely to complete questionnaires. ADR profiles of women and treatment with SSRI were slightly overrepresented in the statistical analysis, which limits its transferability to men and other substance classes. Additionally, pharmacogenetic factors seem to influence study adherence as rapid CYP metabolizers were underrepresented in the evaluation. It is unclear whether the lower participation rate in the questionnaire is due to a higher or lower burden of ADR.

Exposure to AD treatment was recorded as either >14 days or ≤14 days. Exactly time-dependent differences in reported ADR may have been underestimated due to misclassification bias. Furthermore, the applied Haldane-Anscombe correction can lead to biased estimates in small samples, so measures should be interpreted with caution [72].

A strength of the study is its naturalistic design, including a broad age range and heterogeneous antidepressant exposure, which reflects routine clinical practice. Despite the small sample, the data are valuable because of the good gene panel using modern CNV analysis and considering interacting factors. ADR rating was done by the patients, which is a limitation of the results. Future studies should use self and observer ratings to verify the results.

Not all CYP phenotypes were represented. SM were overrepresented due to PC and lower study adherence in RM. There were no UM for CYP2D6 and only three CYP 2C19/2D6 NM/NM as controls. The influence of PC on functional CYP enzyme status has rarely been considered in previous studies, but it is crucial for interpreting study results on PGx-related ADR. Some inhibitors, such as promethazine, require a clear FDA statement of inhibition strength for accurate phenotype calculation. Standardized TDM data were not available, as they were not routinely obtained within the study framework.

This pilot study addresses a largely unexplored area in which larger datasets are currently unavailable. The limited sample size resulted in insufficient power to detect small to moderate effects and restricts the generalizability of the findings. Accordingly, non-significant results from the exploratory regression analyses should be interpreted with caution. The results offer preliminary evidence suggesting an association between ADR and combined CYP 2C19/2D6, considering both psychiatric and non-psychiatric medication. Combined divergent CYP 2C19/2D6 enzyme status indicated association with increased ADR.

These results provide a basis for larger cohort studies to provide sufficient evidence for generating phenotype-specific cross-tabulations.

## Conclusion

The complex pharmacokinetic interaction of the CYP2C19 and CYP2D6 functional status is relevant for the intolerance of AD. A combined nNM enzyme status seems to be associated with a significantly higher ADR risk than a combined NM enzyme status. One potential cause is the alteration of exposure to metabolites and a shift in the metabolite-to-parent drug ratio. AD treatment with less pronounced dependence on CYP2C19 or CYP2D6 metabolism may be considered. This hypothesis requires systematic evaluation in future studies.

This study highlights the need for larger controlled trials to identify risk variants for drug-specific subgroups and provide further evidence for PGx guidelines for AD. Implementing genetic information into clinical decision-making may enhance the safety of AD therapy.

## Supplementary information

Supplementary Tables S1 + S2

## Data Availability

The datasets generated during and/or analysed during the current study are available from the corresponding author upon request.
