# Supplementary material for: Influence of combined CYP2C19 and CYP2D6 phenotypes on adverse drug reactions in patients with major depressive disorder: a clinical cohort study
Source: Pharmacogenomics J. 2026 Apr 9;26(2):13. doi: 10.1038/s41397-026-00407-3 (PMC13065475; doi:10.1038/s41397-026-00407-3)
Supplement: Supplementary file 1 — Supplementary Tables S1 + S2 [file 41397_2026_407_MOESM1_ESM.docx]

**SUPPLEMENTARY INFORMATION**

**Supplementary Table S1**

| **Variable** | **Coefficient (****β)** | **Std. Error** | **t-value** | **p-value** | **95% CI** |
| --- | --- | --- | --- | --- | --- |
| Intercept | 3.63 | 1.72 | 2.11 | 0.04 | 0.12-7.14 |
| CYP2C19 | 0.28 | 0.56 | 0.50 | 0.62 | -0.86-1.41 |
| Age | 0.04 | 0.04 | 1.08 | 0.29 | -0.04-0.12 |
| Sex | 0.19 | 1.13 | 0.16 | 0.87 | -2.13-2.50 |
| Form of antidepressant treatment | 2.41 | 1.54 | 1.57 | 0.13 | -0.73-5.55 |

Linear regression of ADR on CYP2C19, age, sex, and form of antidepressant treatment (*n* = 35).

CYP2C19 metabolizer status coded as ordinal variables (-2 = PM, -1 = IM, 0 = NM, +1 = RM, +2 = UM). Age in years, sex coded as 0 = female, 1 = male, and form of antidepressant treatment coded as 0 = monotherapy, 1 = combined therapy, were included as covariates.

**Supplementary Table S2**

| **Variable** | **Coefficient (β)** | **Std. Error** | **t-value** | **p-value** | **95% CI** |
| --- | --- | --- | --- | --- | --- |
| Intercept | 3.62 | 1.95 | 1.86 | 0.07 | -0.36-7.61 |
| CYP2D6 | 0.12 | 0.84 | -0.14 | 0.89 | -1.84-1.60 |
| Age | 0.04 | 0.04 | 1.02 | 0.32 | -0.04-0.12 |
| Sex | 0.15 | 1.15 | 0.13 | 0.89 | -2.19-2.50 |
| Form of antidepressant treatment | 2.46 | 1.54 | 1.60 | 0.12 | -0.69-5.61 |

Linear regression of ADR on CYP2D6, age, sex, and form of antidepressant treatment (*n* = 35).

CYP2D6 metabolizer status coded as ordinal variables (-2 = PM, -1 = IM, 0 = NM, +2 = UM). Age in years, sex coded as 0 = female, 1 = male, and form of antidepressant treatment coded as 0 = monotherapy, 1 = combined therapy, were included as covariates.
